# Immune cell senescence and chronic bone diseases: osteoimmune mechanisms and therapeutic perspectives

**DOI:** 10.3389/fimmu.2026.1855772

**Published:** 2026-06-05

**Authors:** Zhi Wen, Min Yu, Siren Li, Fan Zeng, Jing Li, Jiabin Li, Guiping Liang, Kang Wang

**Affiliations:** 1 Orthopedics Department, Hunan University of Medicine General Hospital, Huaihua, China; 2 Department of Classical Chinese Medicine, Huaihua Hospital of Traditional Chinese Medicine, Huaihua, China; 3 Department of Orthopedics, The Affiliated Hospital of Jiangxi University of Traditional Chinese Medicine, Nanchang, Jiangxi, China; 4 Graduate School, Hunan University of Chinese Medicine, Changsha, China; 5 Orthopedics Department, The First Hospital of Hunan University of Chinese Medicine, Changsha, China

**Keywords:** chronic bone diseases, immune cell senescence, immune cells, osteoimmune microenvironment, therapeutic strategies

## Abstract

Immune cell senescence is an important intermediary linking organismal ageing, chronic low-grade inflammation, and disordered bone metabolism. With advancing age, immune cells undergo systemic functional remodeling and exhibit a series of characteristic alterations, including reduced proliferative capacity, skewed differentiation, abnormal migration and homing, impaired phagocytic and clearance functions, and changes in their secretory profile. These changes persistently disrupt the osteoimmune microenvironment and ultimately promote enhanced bone resorption, suppressed bone formation, and deterioration of bone quality. This Review centers on the immunological basis of bone homeostasis and systematically summarizes the major biological features of immune cell senescence, with a particular focus on the key cellular mechanisms through which it drives chronic bone disease. It further analyses its pathological manifestations and disease-specific differences in osteoporosis, osteoarthritis, rheumatoid arthritis, and diabetes-related bone disease. Current evidence indicates that the contribution of immune cell senescence varies across different diseases: its pathogenic association appears to be relatively more direct in osteoporosis and rheumatoid arthritis, whereas in osteoarthritis and diabetes-related bone disease it more often acts as a contributor to inflammatory amplification and microenvironmental deterioration. At present, intervention strategies targeting immune cell senescence mainly focus on modulation of macrophage polarization, immune-mediated clearance of senescent cells, restoration of adaptive immune homeostasis, and MSC-related improvement of the local microenvironment, but overall these approaches remain at the preclinical or early translational stage. Future studies should integrate single-cell sequencing, spatial transcriptomics, and multi-omics approaches to define local immune cell senescence landscapes and establish robust biomarker systems, thereby promoting the transition from mechanistic research to precision intervention in chronic bone diseases.

## Graphical Abstract

**Figure:**
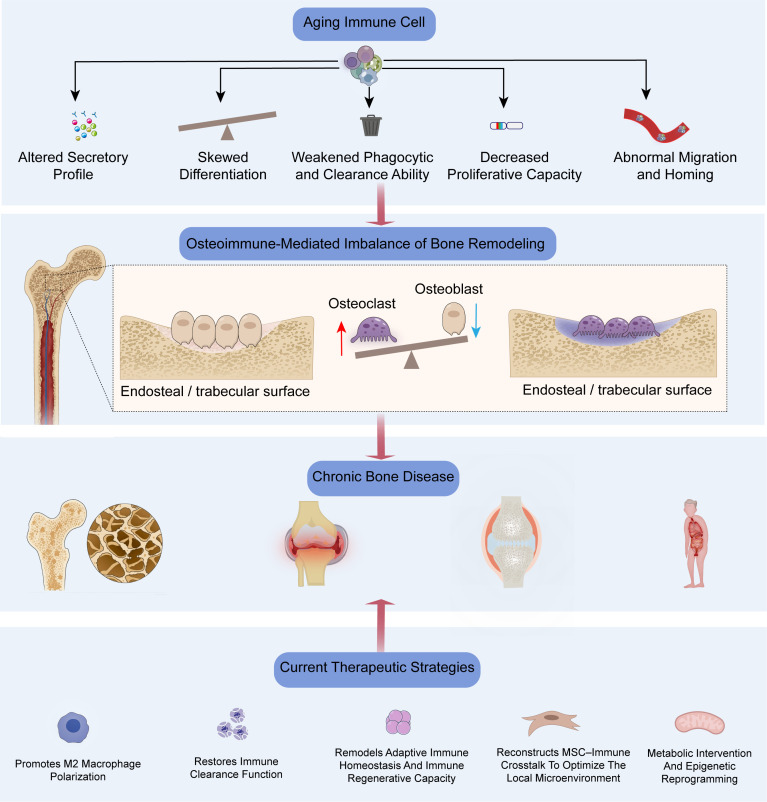
Immune cell senescence is a key link between aging, chronic low-grade inflammation, and bone remodeling imbalance in chronic bone diseases. Aging-driven dysfunction of immune cells reshapes the osteoimmune microenvironment through inflammatory amplification, defective clearance, and altered immune regulation, thereby enhancing osteoclast activity, suppressing osteogenesis, and reducing bone quality. These processes contribute to osteoporosis, osteoarthritis, rheumatoid arthritis, and diabetes-related bone disease with disease-specific patterns and may provide potential targets for precision intervention.

## 1 Introduction

With the acceleration of global population ageing, the incidence of chronic bone diseases continues to rise and has become a major public health concern associated with pain, functional impairment, fractures, and reduced quality of life (1). These disorders have traditionally been attributed to hormonal changes, abnormal mechanical loading, metabolic disturbances, or autoimmune responses. However, accumulating evidence suggests that age-related remodeling of immune function is also a key driver of the onset and progression of chronic bone diseases (2–4). The skeleton is not merely a mechanical support organ, but a dynamic immunometabolic organ that is tightly coupled to the immune system. Within the osteoimmune microenvironment, multiple immune cell populations continuously contribute to the maintenance of bone homeostasis by regulating the RANKL/OPG balance, inflammatory mediator release, and the rhythm of bone remodeling (4–6).

Immunosenescence is a major manifestation of progressive structural and functional remodeling of the immune system during organismal ageing. Its principal features include a decline in naïve T cells, accumulation of memory T cells, imbalanced macrophage polarization, impaired B-cell function, and the persistent presence of chronic low-grade inflammation (7). Unlike simple cellular senescence or inflammaging alone, immunosenescence places greater emphasis on systemic alterations in the overall composition of the immune system, its modes of response, and its capacity for homeostatic regulation (8). In the skeletal system, immunosenescence may not only compromise immune regulation and tissue repair following injury, but also continuously disturb the osteoimmune microenvironment, thereby contributing to disordered bone metabolism and the progression of chronic bone diseases (9).

In recent years, research on osteoimmunology and cellular senescence has advanced rapidly. However, the existing literature has largely focused separately on osteoimmune regulation, senescence of bone cells, or inflammatory mechanisms in individual diseases. A systematic integration of how immunosenescence serves as a key intermediary linking ageing to chronic bone disease is still lacking. Against this background, this Review begins with the immunological basis of bone homeostasis and then outlines the major biological features of immunosenescence. It further focuses on the key mechanisms by which senescence in different immune cell populations drives chronic bone disease, and analyses its pathological manifestations in osteoporosis, osteoarthritis, rheumatoid arthritis, and diabetes-related bone disease. Finally, potential intervention strategies targeting immunosenescence and future translational directions are summarized, with the aim of providing a new theoretical framework for mechanistic studies and precision treatment of chronic bone diseases.

## 2 Immunological basis of bone homeostasis

Bone homeostasis is a physiological process that maintains the dynamic balance between bone formation and bone resorption through the coordinated actions of multiple bone cell types (10, 11). A growing body of evidence indicates that this balance is not maintained solely by bone-related cells in isolation, but is instead profoundly influenced and regulated by the immune system (12, 13). Thus, the skeleton is not only a mechanical support organ, but also a dynamic immunometabolic integrative organ. A complex bidirectional regulatory network exists between the skeletal and immune systems, and this interaction is essential for the maintenance of skeletal health (14–17).

### 2.1 Components of the osteoimmune microenvironment

The skeletal and immune systems are not independent of one another, but are instead closely interconnected through complex cellular and molecular interactions (14, 16–18). The bone marrow is not only the principal site of hematopoiesis, but also a critical microenvironment for the residence, differentiation, and functional activity of multiple immune cell populations (19, 20). Building on this foundation, the skeletal and immune systems establish a tightly interconnected network through shared cells, molecules, and signaling pathways, thereby forming the osteoimmune microenvironment, which serves as a critical regulatory basis for maintaining bone homeostasis. The composition of this microenvironment involves multiple innate and adaptive immune cell populations.

Based on their functional properties, these immune cells can be broadly categorized into several interconnected groups. First, macrophages and monocytes constitute essential components of innate immunity and play pivotal roles in inflammatory regulation, tissue repair, and the maintenance of bone homeostasis (18). Among them, bone marrow-resident macrophages, termed osteal macrophages (OsteoMacs), and CX3CR1^+ monocytes represent key cellular populations involved in the maintenance of bone homeostasis. They participate in local immune surveillance and the regulation of bone remodeling through the phagocytosis of apoptotic debris and the secretion of regulatory factors (14, 21–23). T cells are important regulators linking immune responses to bone remodeling. Different subsets, such as Th17 and Treg cells, exert distinct effects on bone metabolism by modulating RANKL expression and the inflammatory microenvironment (24–26). B cells also exhibit dual regulatory functions, as they can both inhibit osteoclast formation and, under specific conditions, promote bone resorption (27). In addition, other immune cell populations, including neutrophils, natural killer (NK) cells, dendritic cells (DCs), and myeloid-derived suppressor cells (MDSCs), collectively contribute to the composition of the osteoimmune microenvironment. Among them, neutrophils can exacerbate local bone tissue damage in inflammatory bone diseases (28). NK cells primarily participate in the regulation of bone metabolism through the secretion of cytokines such as IFN-γ, whereas MDSCs influence osteoimmune homeostasis through their immunosuppressive functions (29–31).

### 2.2 Basic processes of bone remodeling under homeostatic conditions

Bone remodeling is a continuous and dynamic process involving both bone formation and bone resorption, with the aim of preserving skeletal integrity, adapting to mechanical stress, and regulating mineral balance (19, 32, 33). This process is primarily accomplished through the coordinated actions of several key cell types. During bone remodeling, osteoblasts (OBs) are responsible for the synthesis and mineralization of bone matrix and are the principal cells mediating bone formation (32, 33). They are derived from mesenchymal stem cells and undergo the classical osteogenic differentiation program, producing osteoid that is subsequently mineralized into mature bone tissue (34). By secreting a range of cytokines, OBs have an essential role in maintaining skeletal health (35). In contrast, osteoclasts (OCs) act as the bone-resorbing cells of the skeleton and are responsible for bone resorption (32, 33, 36). OCs arise from the monocyte/macrophage lineage and are multinucleated cells that dissolve the bone matrix through the secretion of acidic substances and proteolytic enzymes, such as cathepsin K (36). OC activity is regulated by multiple factors, including the RANKL/RANK/OPG system, interactions with OBs, and the Wnt signaling pathway (37–39). In addition to OBs and OCs, osteocytes are also key cells in the regulation of bone remodeling. Osteocytes are differentiated from OBs and become embedded within the bone matrix, where they represent the most abundant cell type in bone tissue. As the principal mechanosensors of bone, they are able to detect changes in mechanical loading and participate in bone remodeling by secreting regulatory molecules such as sclerostin, thereby guiding the adaptive remodeling of the skeleton (40, 41). Bone marrow stromal cells (BMSCs) are also important components of the osteoimmune microenvironment. As progenitor cells for OBs, chondrocytes, and adipocytes, they not only serve as the cellular source of multiple bone-related cell types, but also participate in the regulation of the microenvironment and immune responses through the secretion of cytokines and other mediators (42–44).

From the perspective of the cellular and molecular regulatory mechanisms underlying bone remodeling, the maintenance of bone homeostasis depends on a highly coordinated process of bone turnover between OBs and OCs (45, 46). This process is functionally balanced mainly through multiple pathways, including the RANKL/RANK/OPG axis, the NF-κB pathway, and S1P and Wnt signaling pathways (45, 47). Among these regulatory mechanisms, the RANKL/RANK/OPG axis is considered the core molecular pathway linking bone formation and bone resorption. RANKL binds to the RANK receptor on osteoclast precursors, thereby stimulating osteoclast formation, differentiation, and activation (45, 48, 49)Osteoprotegerin (OPG), in contrast, is a soluble decoy receptor secreted by OBs and osteocytes, which competitively binds RANKL and thus inhibits its interaction with RANK, ultimately suppressing OCs activity (50, 51). Accordingly, the RANKL/OPG ratio is widely regarded as an important indicator of the level of bone resorption and the state of bone metabolic balance (52, 53). Notably, bone remodeling is not a process completed by bone cells alone, as immune cells can also regulate this process through the secretion of specific mediators.

### 2.3 How immune cells regulate bone homeostasis

Immune cells regulate bone formation and bone resorption mainly through the secretion of cytokines and chemokines, as well as through direct cell–cell contact (54, 55). Among them, macrophages have important roles in the maintenance of bone homeostasis and tissue repair, and can influence the functional state of OBs and osteoclast precursors by secreting a variety of regulatory factors (15, 56). T cells are key regulators linking immune responses to bone remodeling, and different subsets participate in the regulation of bone metabolism by modulating RANKL expression and the local inflammatory milieu (24, 25, 55). B cells likewise have regulatory effects on bone metabolism and contribute to the maintenance of the RANKL/RANK/OPG-related balance (27, 48). In addition, myeloid progenitor cells, which serve as the common precursors of monocytes/macrophages and OCs, are also subject to finely tuned regulation of lineage differentiation by multiple signaling pathways (49, 56). Overall, changes in the composition and functional state of immune cells can markedly influence the dynamic balance between bone formation and bone resorption.

## 3 Immunosenescence: concepts and biological characteristics

### 3.1 The concept of immunosenescence

Immunosenescence refers to the progressive structural and functional remodeling of the immune system that occurs with advancing age, and is characterized by impaired innate and adaptive immune responses, disruption of immune homeostasis, and the persistent presence of chronic low-grade inflammation. At its core, immunosenescence does not represent the isolated ageing of a single immune cell type, but rather a systemic functional alteration involving multiple immune lineages (2, 7, 30). Given that this Review focuses on senescence-related changes in specific immune cell populations, including T cells, macrophages, and B cells, and their effects on bone metabolism, the term immune cell senescence is used hereafter. This process should be distinguished from the concepts of cellular senescence and inflammaging.

**Figure 1:**
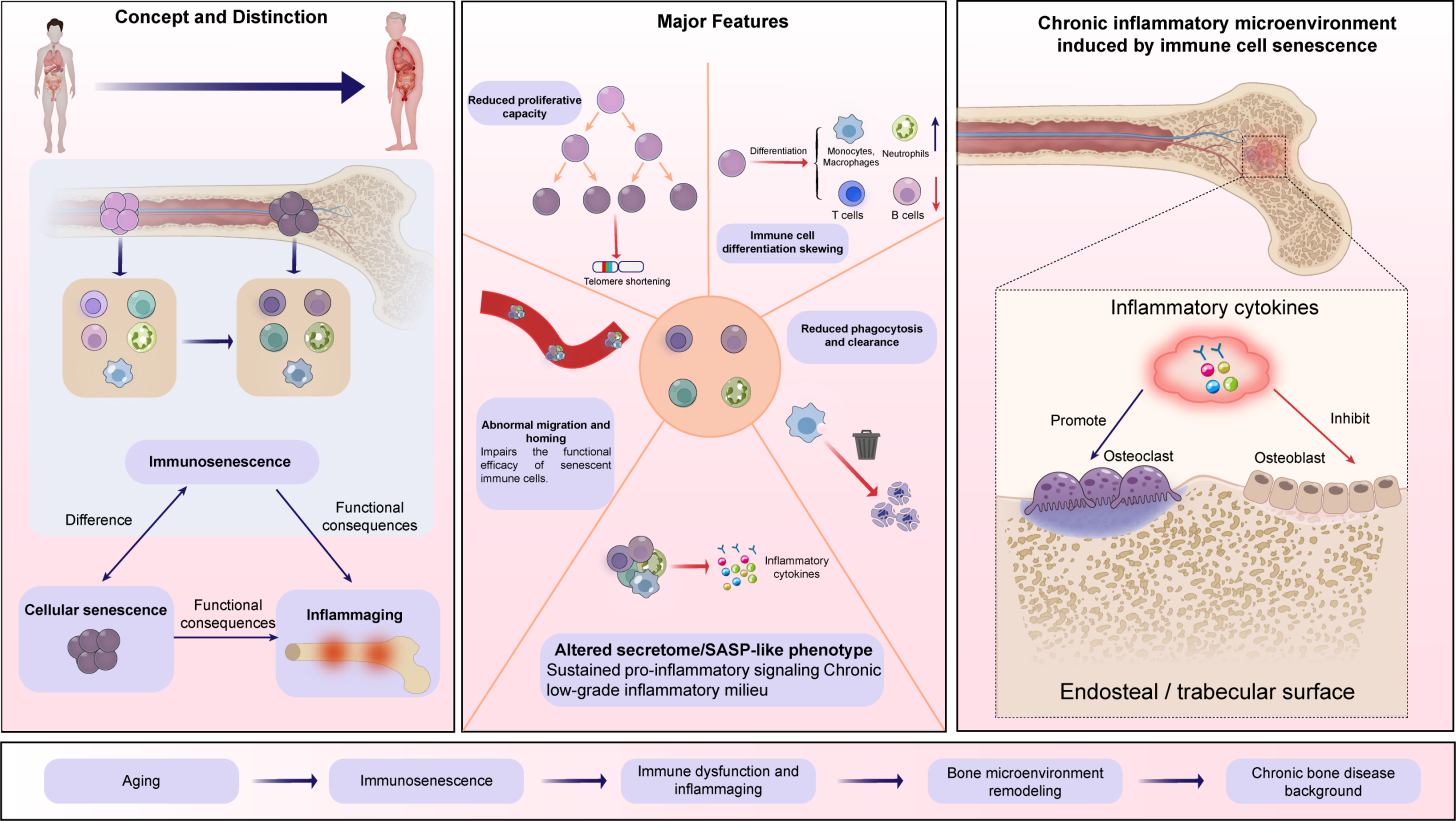
Concept and major biological features of immune cell senescence and its contribution to the pathological background of chronic bone diseases. Immune cell senescence refers to the age-related remodeling of the immune system, characterized by impaired innate and adaptive immune responses, loss of immune homeostasis, and persistent low-grade inflammation. It should be distinguished from cellular senescence, which is defined as stable and irreversible cell-cycle arrest accompanied by sustained metabolic activity and senescence-associated secretory phenotype (SASP), and from inflammaging, which mainly refers to the chronic low-grade pro-inflammatory state associated with aging. The major features of immune cell senescence include reduced proliferative capacity, differentiation bias, impaired migration and homing, defective phagocytosis and clearance, and altered secretory profiles with SASP-like inflammatory activation. Together, these changes reshape the osteoimmune microenvironment toward persistent inflammation, enhanced osteoclastogenesis, and suppressed osteogenesis, thereby establishing a common pathological basis for chronic bone diseases.

Immune cell senescence is characterized by a decline in innate and adaptive immune responsiveness, reduced efficiency of pathogen clearance, and attenuated vaccine responses, together with impaired self-tolerance and the development of a chronic low-grade inflammatory state (57, 58). It does not represent an isolated alteration in a single immune cell type, but rather a process of systemic functional dysregulation involving multiple lineages, including T cells, B cells, NK cells, and myeloid cells. At its core is the persistent remodeling and imbalance of immune function, as reflected by the accumulation of memory T cells, contraction of the naïve T-cell pool, defects in B-cell class switching, and shifts in macrophage polarization (8, 57). In contrast, cellular senescence refers to a stable and irreversible state of cell-cycle arrest that is induced by various stressors, including DNA damage, telomere shortening, and oncogene activation (59–61). Although senescent cells cease to proliferate, they remain metabolically active and display multiple characteristic features, including increased expression of cell-cycle inhibitory proteins, positivity for senescence-associated β-galactosidase (SA-β-gal), and the development of a senescence-associated secretory phenotype (SASP) (62, 63). Cellular senescence has certain physiological significance at early stages, but its long-term accumulation can drive chronic inflammation and contribute to the development of various diseases (60, 64). Inflammaging, by contrast, is generally regarded as one of the functional consequences of immune cell senescence and mainly refers to the persistent, low-grade, systemic pro-inflammatory state present in aged individuals in the absence of overt infection or acute injury (8, 65). This state is driven by senescent immune cells, particularly certain subsets of macrophages and senescent T cells, as well as by SASP factors released from accumulated senescent cells. These SASP components include a wide range of pro-inflammatory cytokines, such as IL-6, TNF-α, and IL-1β, together with chemokines, which persistently act on both local and systemic microenvironments, thereby aggravating tissue damage and promoting the onset and progression of multiple age-related diseases (65–67). Overall, immune cell senescence emphasizes the global functional decline and remodeling of the immune system and is characterized by a series of features associated with impaired immune function. Collectively, these changes provide an important basis for its reshaping of the osteoimmune microenvironments (**Figure 1**).

### 3.2 Major characteristics of immune cell senescence

Senescent immune cells exhibit a series of characteristic alterations, including reduced proliferative capacity, skewed differentiation, abnormal migration and homing, impaired phagocytic and clearance functions, and changes in their secretory profile. Together, these changes contribute to the overall decline of the immune system and the remodeling of the osteoimmune microenvironment (30, 68).

First, reduced proliferative capacity is one of the hallmarks of immune cell senescence. Under conditions of persistent antigenic stimulation or repeated rounds of replication, immune cells may progressively reach the limit of replicative senescence as telomeres gradually shorten, leading to a marked decline in proliferative potential (57). Second, skewed differentiation is also a typical feature of immune cell senescence. With advancing age, hematopoietic stem cells (HSCs) gradually exhibit a myeloid bias, resulting in increased production of myeloid cells such as neutrophils and macrophages, together with reduced output of lymphoid cells such as T cells and B cells, thereby impairing the replenishment capacity of the adaptive immune compartment (8). In addition, the composition of T-cell and B-cell subsets also changes with ageing, as reflected by the accumulation of memory T cells, contraction of the naïve T-cell pool, and impaired germinal center responses in B cells, which in turn reduce the generation of high-affinity antibodies (8, 68). At the same time, abnormalities in migration and homing further compromise the functional effectiveness of senescent immune cells. Under senescent conditions, the migratory and homing capacities of immune cells become dysregulated. For example, dysfunction of the CXCR4/CXCL12 axis may reduce lymph node residence while increasing bone marrow infiltration, thereby affecting the localization of immune cells within specific microenvironments and impairing their normal functions (6). In addition to the changes described above, impaired phagocytic and clearance capacity is another important feature of immune cell senescence. Macrophages, for example, may exhibit reduced FcγR expression, disrupted autophagic flux, and diminished clearance of apoptotic cells during ageing, thereby leading to the persistent accumulation of damaged components and inflammatory stimuli, which further aggravates tissue injury and chronic inflammation (8). Finally, alterations in the secretory profile and the enhancement of a SASP-like pro-inflammatory phenotype represent another major manifestation of functional imbalance in senescent immune cells. Senescent T cells, macrophages, and other immune cell populations can adopt a SASP-like secretory phenotype, continuously releasing pro-inflammatory cytokines such as IL-6, TNF-α, and IL-1β, as well as chemokines and matrix metalloproteinases, thereby promoting a chronic low-grade inflammatory milieu (67, 68) (**Figure 1**).

### 3.3 How immune cell senescence shapes the pathological context of chronic bone disease

On the basis of the features described above, immune cell senescence drives the osteoimmune balance away from homeostatic maintenance towards a pro-inflammatory and pro-osteoclastogenic state through enhanced SASP-like secretion, impaired immune clearance, and persistent inflammatory conditioning of the osteoimmune microenvironment, thereby providing a common pathological background for the onset and progression of chronic bone diseases (2, 3, 6, 60). Senescent immune cells can continuously secrete SASP factors, which further induce osteoblasts, osteoclasts, and osteocytes within bone tissue to enter a senescent state. These senescent bone cells likewise produce SASP factors, thereby forming a vicious cycle (69, 70). In addition, the ability of senescent immune cells to clear other senescent bone cells is impaired, leading to the accumulation of senescent bone cells within bone tissue and further amplifying local inflammation and bone metabolic dysfunction (8, 63, 64). Together, immune cell senescence and inflammation drive the osteoimmune microenvironment towards a pro-osteoclastogenic state. Pro-inflammatory cytokines directly or indirectly regulate the proliferation and differentiation of osteoclast precursors and prolong osteoclast survival. Meanwhile, these inflammatory mediators may disrupt the balance of bone metabolism through multiple signaling pathways, resulting in markedly enhanced bone resorption and suppressed bone formation (6, 66) (**Figure 1**).

## 4 Key cellular mechanisms by which immune cell senescence drives chronic bone disease

The onset and progression of chronic bone diseases are complex processes, in which the systemic remodeling of the osteoimmune microenvironment induced by immune cell senescence has a central driving role. This remodeling is characterized by adaptive immune imbalance, amplification of inflammation, and defective clearance, ultimately giving rise to a pro-inflammatory, pro-osteoclastogenic network that leads to bone loss and microarchitectural deterioration.

### 4.1 T-cell senescence: from phenotypic remodeling to enhanced pro-osteoclastogenic signaling

**Figure 2:**
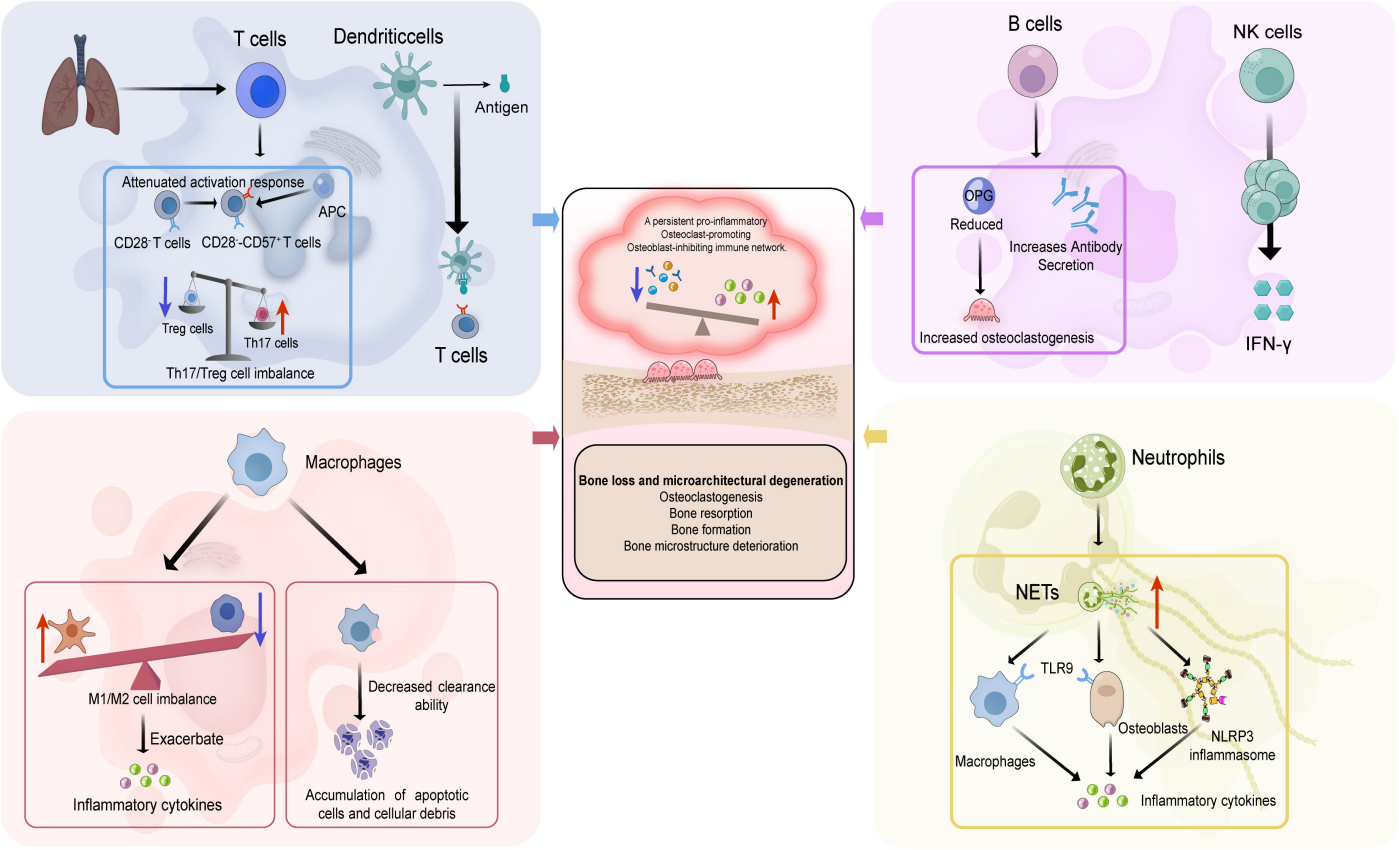
Key cellular mechanisms by which immune cell senescence drives chronic bone diseases. Immune cell senescence remodels the osteoimmune microenvironment and promotes chronic bone diseases by sustaining inflammation, enhancing osteoclastogenesis, and impairing tissue homeostasis. Senescent T cells drive Th17/Treg imbalance, whereas senescent macrophages promote M1-skewed inflammation and defective clearance. Senescent B cells, dendritic cells, neutrophils, and NK cells further disrupt bone immune balance through reduced OPG production, altered antigen presentation, increased NET formation, and changes in IFN-γ-related signaling. Collectively, these alterations establish a pro-inflammatory and pro-osteoclastogenic network that promotes bone loss and microstructural deterioration.

T-cell senescence is one of the most representative alterations during immune cell senescence (71, 72). With advancing age, the thymus undergoes progressive involution, leading to a marked decline in the output of newly generated naïve T cells (72), accompanied by reduced T-cell receptor (TCR) diversity. At the same time, chronic antigenic stimulation and insufficient thymic output increase the proportion of memory/effector T cells in the peripheral blood (72). During repeated rounds of proliferation, a subset of memory T cells develops a senescent state characterized by telomere shortening and functional exhaustion (71). Among these alterations, the loss of the co-stimulatory molecule CD28 is a hallmark of T-cell senescence. CD28^-^ T cells are frequently accompanied by CD57^+^ expression, and these changes weaken T-cell responsiveness to activation signals from antigen-presenting cells (APCs), rendering them more prone to entering a state of anergy or aberrant activation (73). In addition, senescent T cells commonly exhibit mitochondrial dysfunction, characterized by reduced mitochondrial membrane potential, accumulation of reactive oxygen species (ROS), and disturbances in energy metabolism, which further impair their differentiation and immunoregulatory functions (72). At the functional level, senescent T cells maintain a state of low-grade inflammation within the osteoimmune microenvironment through the release of various cytokines and chemokines, and directly or indirectly promote osteoclastogenesis (73–75). Specifically, aging is associated with an increased tendency toward Th17 cell polarization and elevated IL-17 secretion, whereas the number and/or suppressive function of regulatory T cells (Tregs) declines, resulting in Th17/Treg imbalance. The expanded Th17 population produces IL-17 and TNF-α, which act on osteoblasts, synovial fibroblasts, and bone marrow stromal cells to strongly induce RANKL expression through activation of the NF-κB and MAPK signaling pathways, thereby providing critical driving signals for the differentiation and maturation of osteoclast precursors (76–78). In contrast, impairment of Treg function weakens the negative regulation of RANKL signaling, including reduced secretion of IL-10 and TGF-β, thereby further amplifying the pro-osteoclastogenic effects within the osteoimmune microenvironment (76, 78, 79). Meanwhile, CD8^+^ T cells gradually acquire a terminally differentiated phenotype characterized by CD57^+^CD28^-^ expression. Although their cytotoxic activity is diminished, they continuously secrete IFN-γ and TNF-α, thereby cooperatively sustaining local inflammation and enhancing osteoclast activity (72). Notably, a bidirectional relationship exists between T-cell senescence and chronic inflammation, as the persistent inflammatory milieu driven by Th17/Treg imbalance can, in turn, accelerate the progression of T-cell senescence, thereby forming a self-reinforcing vicious cycle. Taken together, T-cell senescence is not merely a simple phenotypic alteration; rather, by reshaping cytokine networks, disrupting Th17/Treg balance, and ultimately upregulating RANKL signaling, it constitutes a key immunological basis underlying the enhanced pro-osteoclastogenic tendency observed in chronic bone diseases (**Figure 2**).

### 4.2 Macrophage senescence: polarization imbalance, impaired clearance, and inflammatory amplification

Macrophages are key cells linking immune inflammation to the regulation of bone homeostasis (76, 80), and the effects of their senescence are mainly reflected in polarization imbalance and impaired clearance function. With advancing age, senescent macrophages become more prone to shift towards a pro-inflammatory M1 phenotype (81–83). They continuously secrete pro-inflammatory mediators such as TNF-α, IL-1β, and IL-6, thereby enhancing osteoclastogenic activity and promoting bone resorption (66, 83). At the same time, senescent macrophages exhibit impaired phagocytic and clearance capacity, leading to the accumulation of apoptotic cells and cellular debris within tissues, which further promotes the spread of inflammation and microenvironmental damage (81, 82). In the context of chronic bone disease, the significance of this change lies in the fact that macrophage senescence is not only a source of pro-inflammatory cytokines, but also an important contributor to the failure of inflammation resolution. Therefore, macrophage senescence represents a critical node in the transition from an acute response to persistent dysregulation of bone metabolism in chronic bone disease (**Figure 2**).

### 4.3 The impact of senescence in other immune cell populations

In addition to T cells and macrophages, the senescence of B cells, DCs, and neutrophils also contributes to the development of chronic bone disease. During aging, the ability of B cells to produce OPG declines, resulting in a reduced OPG/RANKL ratio and the removal of inhibitory constraints on osteoclastogenesis. At the same time, senescent B cells may produce increased levels of autoantibodies, thereby exacerbating local inflammation and bone destruction, a phenomenon that is particularly prominent in rheumatoid arthritis (78, 84). Senescence of DCs is mainly characterized by impaired antigen-presenting capacity, which may indirectly disrupt the stability of the osteoimmune microenvironment by affecting T-cell activation (76). Neutrophil senescence is closely associated with excessive formation of neutrophil extracellular traps (NETs). In aged individuals, impaired neutrophil homing and clearance functions lead to abnormal NET accumulation, and NET-associated components, such as histones and proteases, can promote IL-1β release through activation of TLR9 and the NLRP3 inflammasome, thereby amplifying inflammation and enhancing osteoclast differentiation (85, 86). In addition, senescence of natural killer (NK) cells also warrants attention. Although the number of NK cells may increase with aging, their cytotoxic function declines and their IFN-γ secretion profile becomes altered, potentially contributing indirectly to the remodeling of the osteoimmune microenvironment through modulation of the RANKL/OPG balance or interactions with other immune cells (7, 29). Taken together, senescence across multiple immune cell populations does not represent a set of isolated pathological events, but rather constitutes a sustained immune network that is pro-inflammatory, pro-osteoclastogenic, and anti-osteogenic, thereby providing a microenvironmental basis for the long-term progression of chronic bone disease (**Figure 2**).

## 5 Pathological manifestations of immune cell senescence in representative chronic bone diseases

The effects of immune cell senescence on the skeletal system show certain common features, mainly including the persistent presence of chronic low-grade inflammation, disruption of osteoimmune microenvironmental homeostasis, and impairment of the balance of bone remodeling. However, the dominant cell populations, key pathways, and pathological outcomes of these changes are not entirely identical across different chronic bone diseases. Therefore, it is necessary to further analyze, in the context of specific disease entities, the pathological characteristics and disease-specific differences of immune cell senescence in osteoporosis, osteoarthritis, rheumatoid arthritis, and diabetes-related bone disease.

### 5.1 Osteoporosis

**Figure 3:**
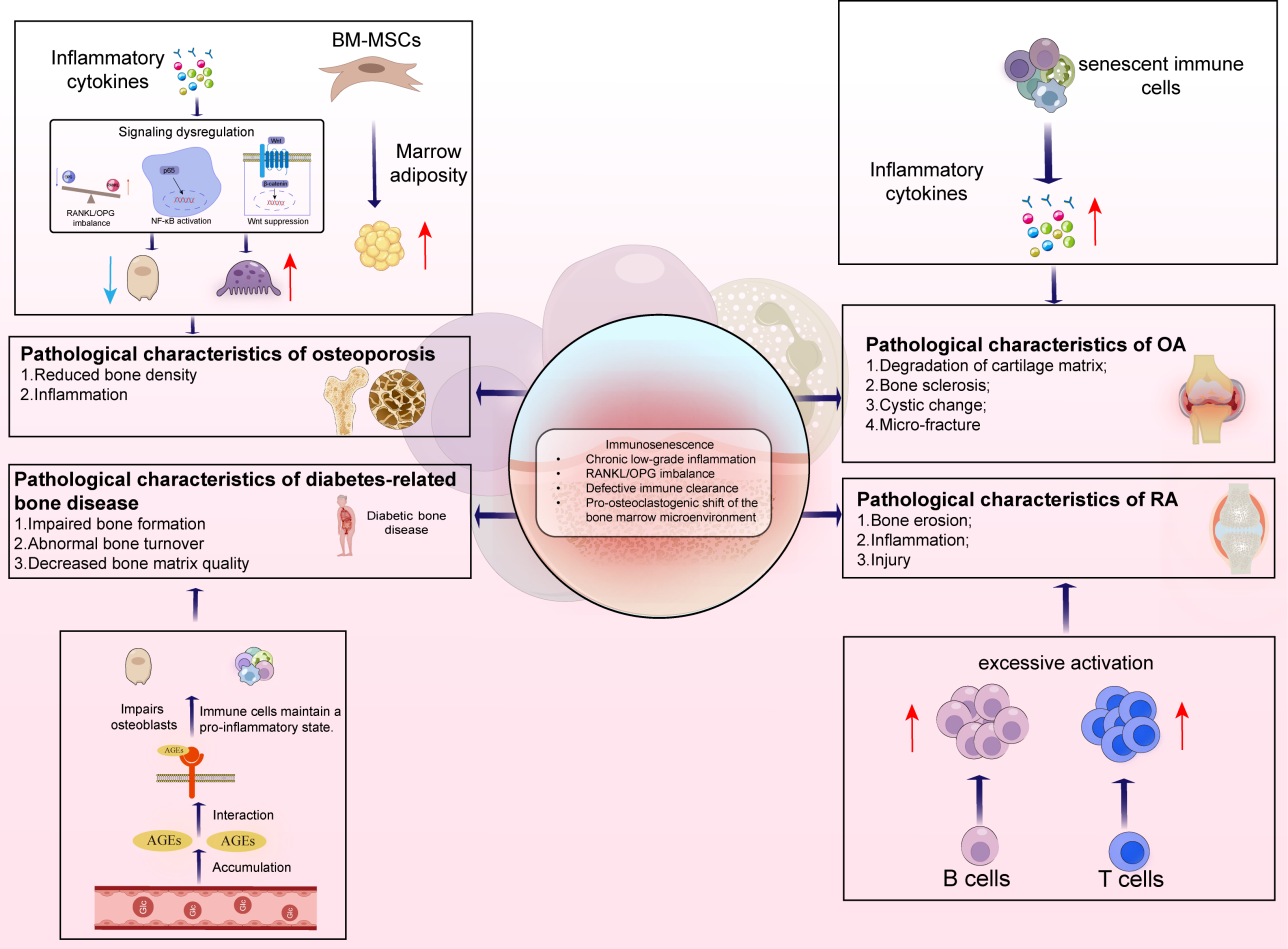
Pathological manifestations of immune cell senescence in representative chronic bone diseases. Immune cell senescence contributes to the development and progression of chronic bone diseases through persistent low-grade inflammation, disruption of osteoimmune homeostasis, impaired immune clearance, and a bone marrow microenvironment favoring osteoclastogenesis, although the dominant mechanisms and pathological outcomes differ among diseases. In osteoporosis, it is mainly associated with RANKL/OPG imbalance, suppressed osteogenesis, enhanced osteoclast activity, and increased marrow adipogenesis. In osteoarthritis, it promotes local inflammatory amplification, cartilage matrix degradation, and abnormal subchondral bone remodeling. In rheumatoid arthritis, premature senescence and aberrant activation of T and B cells amplify synovial inflammation and RANKL-mediated osteoclastogenesis, thereby accelerating bone erosion and joint destruction. In diabetes-related bone disease, chronic hyperglycemia and AGEs–RAGE signaling induce a persistent pro-inflammatory state, impair osteoblast function, disturb bone turnover, and reduce bone matrix quality.

In osteoporosis, immune cell senescence is mainly characterized by an imbalanced RANKL/OPG ratio, suppressed osteogenic signaling, and enhanced adipogenic differentiation. Age-related inflammatory conditions can stimulate osteoblasts and stromal cells to upregulate RANKL while inhibiting OPG production, thereby enhancing osteoclast differentiation and activation (6, 85). At the same time, inflammatory mediators impair osteoblast differentiation and bone formation by activating the NF-κB pathway and suppressing Wnt signaling (66). In addition, senescent T cells, B cells, and macrophages collectively remodel the osteoimmune microenvironment and promote the differentiation of bone marrow mesenchymal stem cells into adipocytes, further compromising osteogenic potential (2). Meanwhile, macrophage polarization becomes imbalanced, leading to the sustained release of inflammatory mediators and further aggravation of bone loss (87). Thus, in osteoporosis, immune cell senescence is not merely an accompanying phenomenon, but is more likely to represent one of the key mechanisms driving RANKL/OPG imbalance, bone marrow adipogenesis, and the establishment of a pro-inflammatory microenvironment. Notably, estrogen deficiency, one of the central driving factors of postmenopausal osteoporosis, can profoundly influence the osteoimmune microenvironment by regulating T-cell and macrophage functions as well as the RANKL/OPG balance, and may exert synergistic effects with immune cell senescence (27, 48) (**Figure 3**).

### 5.2 Osteoarthritis

Osteoarthritis is not merely a degenerative disease characterized predominantly by cartilage degeneration, but rather a whole-joint disorder involving abnormal remodeling of the synovium, subchondral bone, and the local immune microenvironment. In this process, persistent activation of synovial macrophages and amplification of local inflammation are considered to be closely associated with disease progression. Senescent or dysfunctional macrophages can secrete IL-1β, TNF-α, and various matrix metalloproteinases, thereby not only promoting cartilage matrix degradation but also influencing adjacent subchondral bone remodeling by altering the local inflammatory milieu (88, 89). Unlike osteoporosis, osteoarthritis (OA)-associated subchondral bone changes are more commonly characterized not by simple bone loss, but by abnormal remodeling and mechanical imbalance (90). In the early stage of the disease, the subchondral bone may undergo compensatory sclerosis. As the imbalance in bone turnover persists, microarchitectural disorganization, cystic changes, and microfractures may subsequently develop (91). In this context, immune cell senescence may promote the continued progression of local joint pathology through SASP-like secretion, amplification of inflammation, and disruption of the osteoblast–osteoclast balance (89). However, it should be noted that the more substantial evidence in the OA field currently relates mainly to local cellular senescence and inflammaging within the joint, whereas direct evidence supporting immune cell senescence as an independent central driver remains relatively limited. Existing studies more strongly support a role for immune cell senescence in OA progression through the amplification of synovial inflammation and abnormal subchondral bone remodeling, whereas whether it acts as a disease-initiating factor or a secondary amplifying factor still requires further clarification (**Figure 3**).

### 5.3 Rheumatoid arthritis

Rheumatoid arthritis (RA) is a representative disease in which autoimmune dysregulation directly drives bone erosion and is also one of the disorders for which the evidence for immune cell senescence is relatively substantial (92). In RA, immune abnormalities are manifested not only by excessive activation of T cells and B cells, but also by the premature onset of immune cell senescence, most notably in CD4^+ T cells (92, 93). These cells exhibit premature telomere shortening, reduced DNA repair capacity, and mitochondrial dysfunction, together with disrupted lysosomal homeostasis and persistent endoplasmic reticulum stress, gradually acquiring the features of pathogenic effector cells characterized by high TNF secretion, tissue invasiveness, and sustained pro-inflammatory activity (94–96). At the same time, age-associated B cells expand and participate in autoantibody production, antigen presentation, and amplification of inflammation, whereas myeloid cell ageing can also promote the maintenance of a highly metabolic, pro-inflammatory phenotype in synovial macrophages, together sustaining the chronic inflammatory state of the synovial microenvironment (92, 97). On this basis, activated T cells, particularly T helper 17 (Th17) cells, can induce persistent upregulation of RANKL expression in synovial fibroblasts and bone-related cells (78). Meanwhile, B-cell-mediated autoantibody production and antigen presentation further amplify T-cell responses (98). Together, these processes provide sustained stimulation for the differentiation and maturation of osteoclast precursors, ultimately leading to marginal bone erosion in the joints. In parallel, systemic inflammation can further cause generalized bone loss and increase fracture risk (98, 99). Therefore, immune cell senescence in RA is unlikely to represent a single initiating factor that is entirely independent of the autoimmune process; rather, it more likely serves as an important mechanism that continuously amplifies the RANKL/osteoclastogenic pathway, synovial inflammation, and joint tissue damage on the basis of impaired immune tolerance (**Figure 3**).

### 5.4 Diabetes-related bone disease

Diabetes-related bone disease reflects a complex interplay among metabolic dysregulation, chronic inflammation, and immune cell senescence, and is characterized primarily by impaired bone quality rather than by a simple reduction in bone mass. Under conditions of chronic hyperglycemia, advanced glycation end products (AGEs) accumulate extensively and bind to their receptor, receptor for advanced glycation end products (RAGE) (100, 101). On the one hand, this process directly impairs the proliferation, differentiation, and osteogenic function of osteoblasts. On the other hand, it also induces immune cells such as monocytes/macrophages to maintain a persistent pro-inflammatory state, thereby keeping the osteoimmune microenvironment in a state of chronic low-grade inflammation (102). In this context, immune cell senescence may further contribute to the imbalance of bone remodeling. Persistent inflammatory stimulation helps enhance osteoclast activity, while simultaneously suppressing osteoblast function and disturbing bone matrix homeostasis, ultimately leading to impaired bone formation, abnormal bone turnover, and deterioration of bone matrix quality (102). Therefore, even in the absence of a marked reduction in bone mineral density, patients with diabetes may still exhibit increased bone fragility and a higher risk of fracture as a consequence of impaired bone quality. It should be noted that direct mechanistic evidence for the role of immune cell senescence in diabetes-related bone disease remains relatively limited at present. Existing studies more strongly support its involvement as a contributing factor in hyperglycemia-induced chronic inflammation and osteoimmune microenvironmental imbalance, rather than as a fully established single core pathogenic mechanism(**Figure 3**).

## 6 Potential therapeutic strategies targeting immune cell senescence

Targeting immune cell senescence has emerged as an important avenue for intervention in chronic bone diseases, with the central aim of improving the maintenance of bone homeostasis and tissue repair capacity through remodeling of the osteoimmune microenvironment (30, 103, 104). At present, most of these strategies remain at the preclinical research or early proof-of-concept stage and can be broadly categorized into several directions, including modulation of innate immune cell phenotypes, enhancement of immune clearance of senescent cells, restoration of adaptive immune homeostasis, and improvement of the osteoimmune microenvironment through exosomes, metabolic reprogramming, and epigenetic reprogramming (4, 105, 106). It should be noted that these approaches differ substantially in the strength of evidence, disease applicability, and clinical translatability, and that most are still confined to the preclinical or proof-of-concept stage.

### 6.1 Regulating macrophage polarization to optimize the rhythm of tissue repair

Modulation of macrophage polarization is currently one of the therapeutic strategies with the greatest translational potential in chronic bone disease (107). Macrophages have dual roles in bone tissue repair and the maintenance of osteoimmune homeostasis (108). Persistent M1 skewing aggravates inflammation and impedes repair, whereas promoting a shift towards an M2-like phenotype facilitates the resolution of inflammation, tissue remodeling, and angiogenesis (107, 109). Existing studies suggest that signaling pathways such as NF-κB, STAT1/STAT6, and PPARγ may all serve as potential intervention nodes, and that modulation of these pathways can influence the direction of macrophage polarization (110). Biomimetic M2 macrophage-based approaches and biomaterial delivery strategies have also shown promising preclinical efficacy (111). However, a major limitation of this strategy lies in the fact that macrophage polarization is itself highly stage-dependent and tissue-specific. An excessive emphasis on M2 polarization is not invariably beneficial and may even compromise host defense or promote fibrotic responses (112, 113). Therefore, the key goal in the future is not simply to enhance M2 polarization, but rather to restore dynamic immune balance across different stages of disease.

### 6.2 Restoring immune clearance capacity to enhance the elimination of senescent cells

In youth, immune cells are capable of recognizing and eliminating senescent cells, with macrophages serving as key “scavengers” in this process (114, 115). However, with advancing age, the recognition and cytotoxic capacities of these immune cells decline markedly, allowing senescent cells to evade immune surveillance and accumulate (116). Enhancing the capacity of local macrophages to clear senescent cells is therefore a critical step in promoting bone repair. Current efforts have focused on improving the efficiency with which macrophages eliminate senescent cells by targeting signaling axes involving CX3CR1^+ macrophages, TREM2, DAP12, and complement C3aR (87, 116). Accordingly, strengthening macrophage-mediated recognition, phagocytosis, and efferocytosis of senescent cells has important theoretical value. The central challenge in this area lies not in establishing its conceptual feasibility, but in achieving local, stage-specific, and selective clearance strategies that can balance the demands of tissue repair with immune safety.

### 6.3 Restoring adaptive immune homeostasis and immune regenerative capacity

In addition to innate immunity, interventions targeting T-cell senescence and impaired immune regeneration may also have therapeutic potential. Expansion of regulatory T cells using IL-2/anti-IL-2 complexes may help preserve immune homeostasis and suppress chronic inflammation (7). Strategies that promote thymic regeneration, such as KGF- or IL-22-based interventions, may increase the output of naïve T cells and ameliorate the age-related decline in T-cell diversity (117). Furthermore, the myeloid bias driven by hematopoietic stem cell (HSC) ageing represents an important upstream mechanism of immune cell senescence (117), and approaches such as partial reprogramming may theoretically help restore a more youthful state in HSCs, thereby improving the overall composition of the immune system (118). Compared with strategies that locally regulate the osteoimmune microenvironment, this direction places greater emphasis on systemic immune reconstruction. Its advantage lies in the potential to target upstream mechanisms of immune cell senescence, but it also faces translational barriers, including broad targeting, limited tissue specificity, and the complexity of safety evaluation.

### 6.4 Reconstructing MSC–immune crosstalk to optimize the local microenvironment

The interaction between mesenchymal stem cells (MSCs) and immune cells is a fundamental basis for bone tissue homeostasis and repair. In the context of ageing, functional decline in MSCs, together with disrupted communication between MSCs and immune cells such as macrophages, further compromises the bone regenerative microenvironment. Accordingly, modulation of local immune responses through MSC-derived exosomes or transplantation of healthy MSCs has become an increasingly studied therapeutic direction (119, 120). In particular, exosomes enriched in molecules such as miR-146a have been shown to suppress NLRP3 inflammasome activation, reduce the release of pro-inflammatory mediators, and promote M2 polarization (121, 122). The advantage of this approach lies in its dual potential for immunomodulation and tissue repair. However, its translation remains limited by several challenges, including the lack of standardized manufacturing protocols, insufficient delivery efficiency, unstable tissue homing, and inadequate long-term safety data. Future progress will require support from standardized production systems and disease-specific delivery strategies.

### 6.5 Metabolic intervention and epigenetic reprogramming

Immune cell senescence is commonly accompanied by disordered energy metabolism, NAD^+ depletion, mitochondrial dysfunction, and epigenetic drift. Accordingly, metabolic and epigenetic reprogramming may provide more upstream points of intervention for targeting immune cell senescence. Strategies that increase NAD^+ levels, such as nicotinamide mononucleotide (NMN) or nicotinamide riboside (NR), may improve immune cell function by restoring mitochondrial activity and activating sirtuin (SIRT) pathways (123, 124). At the same time, modulation of epigenetic enzymes such as enhancer of zeste homologue 2 (EZH2) and DNA methyltransferases (DNMTs) may also reverse, at least in part, aberrant senescence-associated gene expression programs (116, 125). However, it should be recognized that these interventions currently remain largely at the stage of theoretical extrapolation or inference from studies in other disease systems, and direct evidence in chronic bone disease is still limited.

Overall, intervention strategies targeting immune cell senescence have evolved into a multi-layered framework encompassing local immunomodulation, senescent cell clearance, adaptive immune remodeling, and metabolic/epigenetic intervention. However, substantial differences remain among these strategies in terms of evidentiary support, applicable settings, and translational feasibility. At the current stage, a key challenge for further progress in this field is how to select the most appropriate interventional approach according to disease type and stage.

### 6.6 Drug repurposing and nutritional and lifestyle interventions

In addition to the strategies discussed above, drug repurposing as well as nutritional and lifestyle interventions also deserve attention. Senolytic combinations represented by dasatinib plus quercetin have demonstrated the ability to selectively eliminate senescent cells, alleviate bone loss, and improve bone quality in multiple animal models of chronic bone diseases (126). A phase II randomized controlled trial in postmenopausal women further showed that intermittent administration increased the levels of bone formation markers and exhibited a trend toward improved bone mineral density in subgroups with a high senescent cell burden (127). Meanwhile, caloric restriction and intermittent fasting may delay immunosenescence and promote bone repair by regulating nutrient-sensing pathways such as mTOR and AMPK and by improving mitochondrial function (128). Probiotics and synbiotic formulations have also shown anti-inflammatory and anti-osteoclastogenic effects in postmenopausal bone loss models through modulation of the gut microbiota–immune axis (129). Nevertheless, the specific targets and long-term safety of these strategies within the osteoimmune microenvironment still require further investigation (**Table 1**).

**Table 1:** Therapeutic strategies targeting immune cell senescence in chronic bone diseases.

Strategy	Key targets	Representative interventions	Main limitations	References
Macrophage polarization regulation	M1/M2 balance; NF-κB; STAT1/STAT6; PPARγ	NF-κB/STAT/PPARγ-targeted agents; biomimetic M2 macrophages; biomaterial-based delivery	Stage- and tissue-dependence; excessive M2 skewing; fibrosis/host-defense risk	(110–113)
Senescent cell clearance enhancement	Senescent cell recognition/efferocytosis; CX3CR1+; TREM2/DAP12; C3aR	CX3CR1+ macrophage targeting; TREM2/DAP12 modulation; complement-related targeting	Selective local clearance is difficult; immune safety concerns	(85, 114–116)
Adaptive immune homeostasis restoration	T-cell senescence; Treg homeostasis; thymic function; HSC aging	IL-2/anti-IL-2 complexes; KGF; IL-22; partial reprogramming	Broad targeting; low tissue specificity; safety complexity	(117, 118)
MSC–immune crosstalk reconstruction	MSCs/exosomes; miR-146a; NLRP3 inflammasome	Healthy MSC transplantation; MSC-derived exosomes; bioactive exosome delivery	Poor standardization; unstable homing; limited long-term safety data	(119–122)
Metabolic/epigenetic reprogramming	NAD+ metabolism; mitochondria; SIRT; EZH2; DNMTs	NMN; NR; epigenetic enzyme modulation	Limited disease-specific evidence; uncertain tissue specificity and long-term efficacy	(123–125)
Drug repurposing and nutritional/lifestyle interventions	Senescent cells; SASP; mTOR/AMPK; mitochondrial function; gut microbiota–immune axis	Dasatinib + quercetin; caloric restriction; intermittent fasting; probiotics/synbiotics	Limited disease-specific clinical evidence; unclear osteoimmune targets; variable individual responses; uncertain long-term safety	(126–129)

## 7 Discussion

Overall, immune cell senescence is not merely an accompanying phenomenon in chronic bone disease, but more likely an important intermediary linking organismal ageing, chronic low-grade inflammation, and disordered bone metabolism. Although senescence in different immune cell lineages displays distinct phenotypes and modes of action, their shared consequence is the persistent disruption of the osteoimmune microenvironment, which drives imbalance in bone remodeling and promotes the progression of chronic bone diseases.

However, the magnitude and pathological significance of immune cell senescence are not entirely consistent across different diseases. In osteoporosis, its association with bone loss is relatively direct and is mainly manifested by chronic inflammation, bone marrow adipogenesis, and sustained enhancement of pro-osteoclastogenic signaling. In rheumatoid arthritis, immune cell senescence acts in close concert with autoimmune activation, and therefore its role in promoting synovial inflammation, RANKL upregulation, and bone erosion is supported by more direct evidence. By contrast, in osteoarthritis and diabetes-related bone disease, the current evidence more strongly supports immune cell senescence as an amplifier of inflammation and a contributor to microenvironmental deterioration during disease progression, whereas whether it constitutes a core driver at the stage of disease initiation still requires further mechanistic investigation. At the same time, recent studies also suggest that immune cell senescence is not simply characterized by weakened immune responsiveness, but may also promote the formation of persistent inflammatory phenotypes in pathogenic T cells and macrophages through dysfunction of the mitochondria, lysosomes, and endoplasmic reticulum.

Several key limitations remain in this field. First, immune cell senescence still lacks unified, stable, and tissue-specific evaluation criteria, and substantial differences exist among studies with respect to phenotypic markers and functional indicators. Second, the available evidence is still derived predominantly from animal experiments and *in vitro* studies, and peripheral blood immune phenotypes cannot fully represent the local status within the bone marrow and bone tissue. Third, most current studies can demonstrate only associations and are still insufficient to clearly distinguish whether immune cell senescence acts as a disease-initiating factor, a progression amplifier, or a secondary phenomenon arising in the setting of chronic inflammation.

At the translational level, targeting immune cell senescence offers a new conceptual framework for the intervention of chronic bone diseases. However, the approaches that are currently closest to practical application still mainly involve local remodeling of the osteoimmune microenvironment, such as modulation of macrophage polarization and MSC-related interventions. By contrast, strategies aimed at restoring immune surveillance, rebuilding adaptive immunity, or achieving metabolic or epigenetic reprogramming, although theoretically promising, remain constrained by limited levels of evidence, insufficient tissue specificity, and uncertain long-term safety. Future studies should further integrate single-cell sequencing, spatial transcriptomics, and multi-omics approaches to define local immune cell senescence landscapes across different chronic bone diseases, and to establish biomarker systems matched to disease stratification and therapeutic evaluation, thereby facilitating the transition from mechanistic understanding to precision intervention.
