# Correction: Immune cell senescence and chronic bone diseases: osteoimmune mechanisms and therapeutic perspectives

**DOI:** 10.3389/fimmu.2026.1905339

**Published:** 2026-06-19

**Authors:** Zhi Wen, Min Yu, Siren Li, Fan Zeng, Jing Li, Jiabin Li, Guiping Liang, Kang Wang

**Affiliations:** 1 Orthopedics Department, Hunan University of Medicine General Hospital, Huaihua, China; 2 Department of Classical Chinese Medicine, Huaihua Hospital of Traditional Chinese Medicine, Huaihua, China; 3 Department of Orthopedics, The Affiliated Hospital of Jiangxi University of Traditional Chinese Medicine, Nanchang, Jiangxi, China; 4 Graduate School, Hunan University of Chinese Medicine, Changsha, China; 5 Orthopedics Department, The First Hospital of Hunan University of Chinese Medicine, Changsha, China

**Keywords:** chronic bone diseases, immune cell senescence, immune cells, osteoimmune microenvironment, therapeutic strategies

In the published article, there was a mistake in the **Funding** statement. One funding source was displayed as “Department of Orthopedics, Hunan University of Medicine General Hospital, Grant No.: 2026JJ80468”. The correct statement is “Hunan Provincial Natural Science Foundation, Grant No.: 2026JJ80468”.

The correct **Funding** statement reads:

“The author(s) declared that financial support was received for this work and/or its publication. This work was supported by the following projects: Administration of Traditional Chinese Medicine of Jiangxi Province, Grant No.: 2024B0198; Jiangxi Provincial Natural Science Foundation, Grant No.: 20252BAC200563; Hunan Provincial Natural Science Foundation, Grant No.: 2026JJ80468.”

The original version of this article has been updated.

